# Supplementary figures and images for: Efficacy and Safety Ablation Index-Guided High-Energy Linear Ablation for Persistent Atrial Fibrillation: PVI Plus Linear Ablation of Mitral Isthmus and Posterior Box Isolation
Source: J Clin Med. 2023 Jan 12;12(2):619. doi: 10.3390/jcm12020619 (PMC9862717; doi:10.3390/jcm12020619)

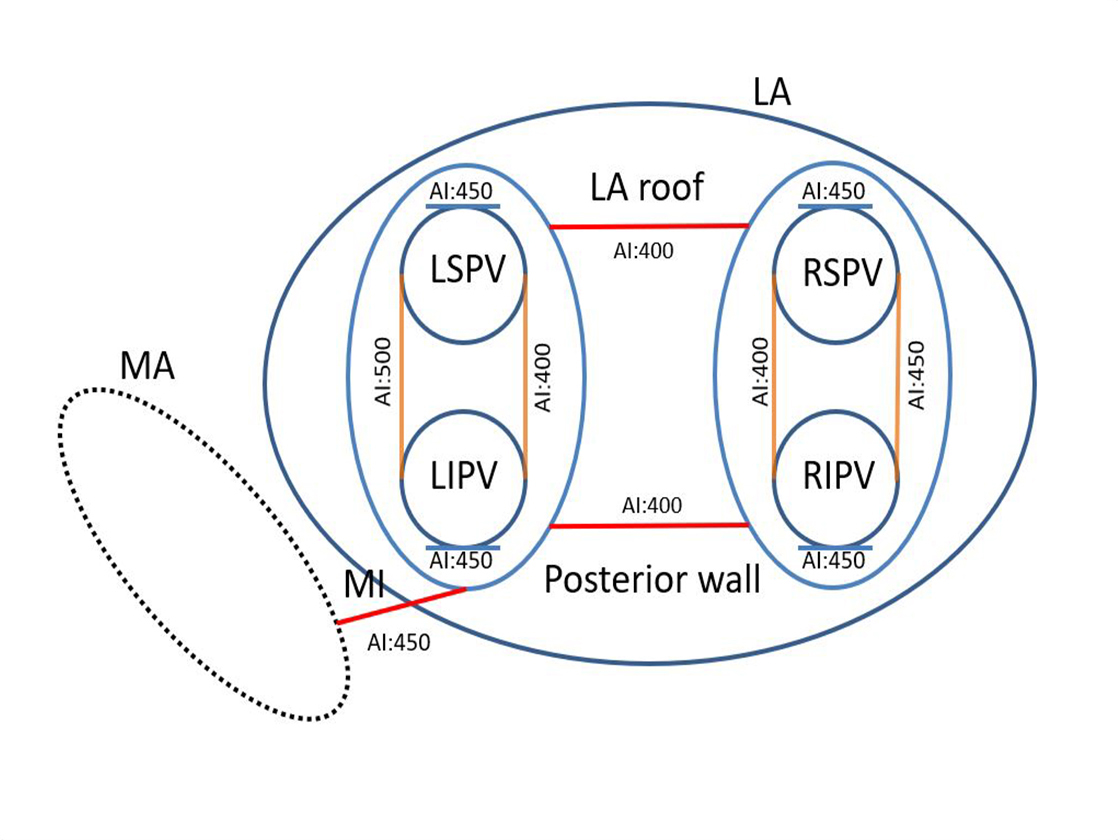

Supplement: Supplementary file 1 [file jcm-12-00619-s001.zip › supplementary- Figure S1.jpg]

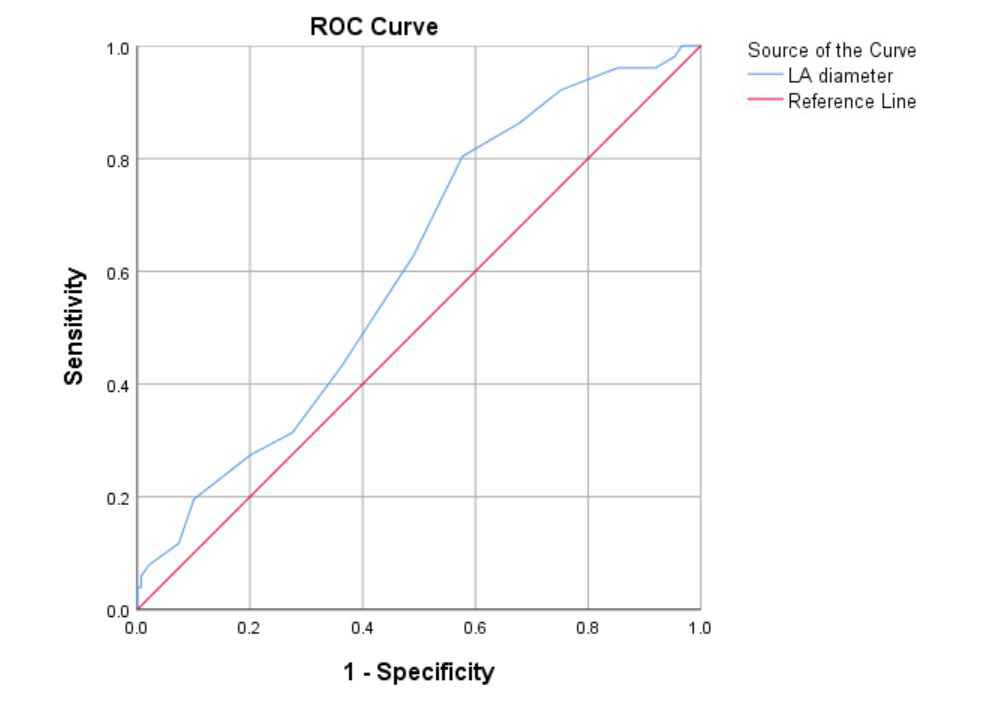

Supplement: Supplementary file 1 [file jcm-12-00619-s001.zip › supplementary- Figure S2.jpg]

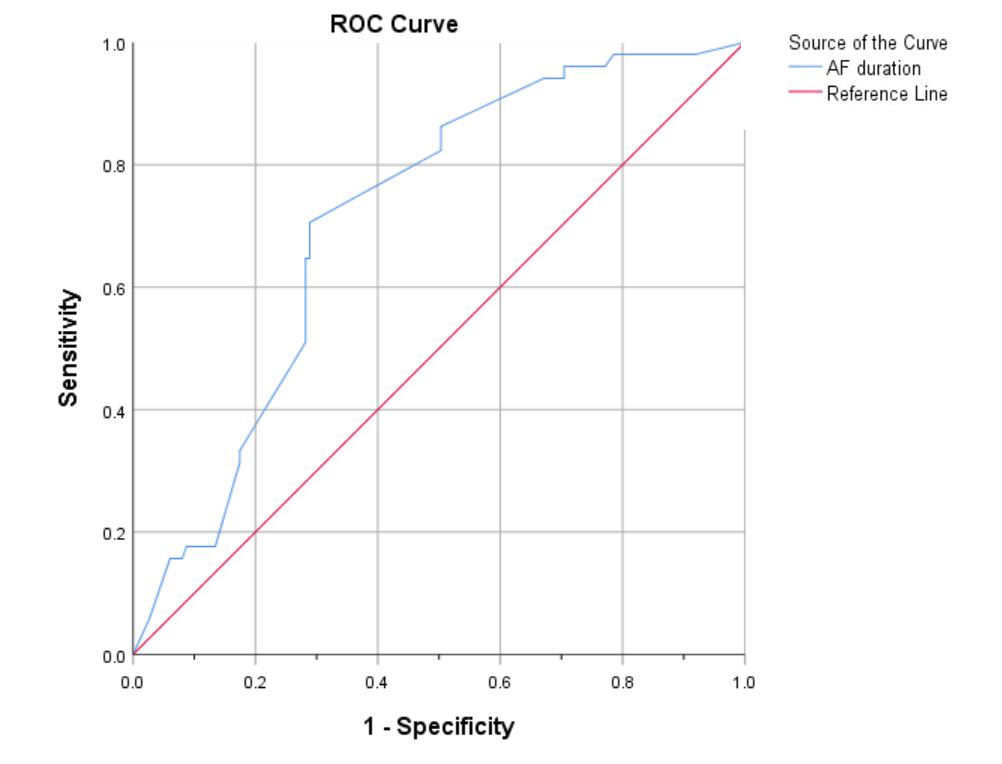

Supplement: Supplementary file 1 [file jcm-12-00619-s001.zip › supplementary- Figure S3.jpg]
